# Universal quantum gates for photon-atom hybrid systems assisted by bad cavities

**DOI:** 10.1038/srep24183

**Published:** 2016-04-12

**Authors:** Guan-Yu Wang, Qian Liu, Hai-Rui Wei, Tao Li, Qing Ai, Fu-Guo Deng

**Affiliations:** 1 Department of Physics, Applied Optics Beijing Area Major Laboratory, Beijing Normal University, Beijing 100875, China; 2 School of Mathematics and Physics, University of Science and Technology Beijing, Beijing 100083, China; 3 State key Laboratory of Low-Dimensional Quantum Physics and Department of Physics, Tsinghua University, Beijing 100084, China

## Abstract

We present two deterministic schemes for constructing a CNOT gate and a Toffoli gate on photon-atom and photon-atom-atom hybrid quantum systems assisted by bad cavities, respectively. They are achieved by cavity-assisted photon scattering and work in the intermediate coupling region with bad cavities, which relaxes the difficulty of their implementation in experiment. Also, bad cavities are feasible for fast quantum operations and reading out information. Compared with previous works, our schemes do not need any auxiliary qubits and measurements. Moreover, the schematic setups for these gates are simple, especially that for our Toffoli gate as only a quarter wave packet is used to interact the photon with each of the atoms every time. These atom-cavity systems can be used as the quantum nodes in long-distance quantum communication as their relatively long coherence time is suitable for multi-time operations between the photon and the system. Our calculations show that the average fidelities and efficiencies of our two universal hybrid quantum gates are high with current experimental technology.

A quantum computer^1^ can run the famous Shor’s algorithm^2^ for integer factorization and implement Grover-Long algorithm^3^,^4^ for unsorted database search. In past decades, it has attracted much attention. Quantum logic gates are the key elements in quantum computers and play a critical role in quantum information processing (QIP). Two-qubit controlled-not (CNOT) gates together with single-qubit gates are sufficient for universal quantum computing^1^,^5^. In 2004, Shende proposed a “small-circuit” structure which is used to construct CNOT gates^6^. In the domain of three-qubit gates, Toffoli gate has attracted much attention and it is universal. Together with Hadamard gates, it can realize unitary manipulation for a multi-qubit system^7^,^8^. Moreover, it plays an important role in phase estimation^1^, complex quantum algorithms^2^,^3^,^4^, error correction^9^, and fault tolerant quantum circuits^10^. In 2009, the optimal synthesis for a Toffoli gate with six CNOT gates was proposed^11^. Up to now, for a general three-qubit logic gate, the optimal synthesis requires twenty CNOT gates^12^, which means that this method increases the difficulty and complexity of experiments and the possibility of errors largely. It is significant to seek a simpler scheme to directly implement multi-qubit gates.

By far, many physical systems have been used to implement quantum logic gates, such as photons in the polarization degree of freedom (DOF)^13^,^14^,^15^ and those in both the polarization and the spatial-mode DOFs (the hyper-parallel photonic quantum computing)^16^,^17^,^18^, nuclear magnetic resonance^19^,^20^,^21^,^22^, quantum dots^23^,^24^,^25^,^26^,^27^, diamond nitrogen-vacancy center^28^,^29^,^30^, superconduting qubits^31^,^32^, superconducting resonators (microwave photons)^33^,^34^, and hybrid quantum systems^35^,^36^. Cavity quantum electrodynamics (QED) is a promising physical platform for constructing universal quantum logic gates as it can enhance the interaction between a photon and an atom (or an artificial atom). Because of the robustness against decoherence, photons are the perfect candidates for fast and reliable long-distance communication. Meanwhile, the stationary qubits are suitable for processor and local storage. Quantum logic gates between flying photon qubits and stationary qubits hold a great promise for quantum communication and computing, especially for quantum repeaters, distributed quantum computing, and blind quantum computing. Wei and Deng^36^ proposed some interesting schemes for universal hybrid quantum gates which use quantum dots inside double-sided optical microcavities as stationary qubits and the flying photon as the control qubit. An atom trapped in an optical microcavity is an attractive candidate for a stationary qubit. The interaction time between a single atom and the cavity in which the atom is trapped can be maintained for 10 s^37^. By using the atoms interacting with local cavities as the quantum nodes and the photon transmitting between remote nodes as the quantum bus, one can set up a quantum network to realize a large-scale QIP.

Many schemes^38^,^39^,^40^,^41^,^42^,^43^,^44^,^45^,^46^ for QIP tasks, assisted by the input-output process in atom-cavity system, have been proposed. Duan and Kimble^41^ proposed a scheme for the construction of a controlled phase-flip (CPF) gate between an atom trapped in a cavity and a single photon. The strong coupling between the atom and the cavity can provide a large Kerr nonlinearity. Combined with the input-output process of the flying single photon, a universal quantum gate can be achieved^41^. Interestingly, the atom-photon coupling in a optical cavity have been implemented in experiments. For example, Reiserer *et al*.^47^ demonstrated an optical nondestructive detection based on reflecting a photon from an optical cavity^41^ containing a single atom in 2013. Tiecke *et al*.^48^ realized a system in which a single atom, trapped in a photonic crystal cavity, switches the phase of a photon and a single photon modifies the phase of an atom in 2014. In the same year, Reiserer *et al*.^49^ implemented a CPF gate between the spin state of a single trapped atom and the polarization state of a photon. Kalb *et al*.^50^ realized a heralded transfer of a polarization qubit from a photon onto a single atom. It is significant for seeking a realization of QIP task in the weak coupling region with a bad cavity. Turchette *et al*.^51^ completed a measurement of conditional phase shifts for quantum logic in an intermediate atom-cavity coupling regime with a bad cavity. Dayan *et al*.^52^ achieved an experiment in which the transport of photons is regulated by one atom trapped in a cavity in an intermediate atom-cavity coupling regime with a bad cavity. Without the requirements of good cavities or strict strong coupling strength, many theoretical QIP tasks have been proposed, such as quantum gates^53^,^54^,^55^,^56^,^57^,^58^,^59^, generation of entangled states^60^, and quantum controlled teleportation^61^. Xiao *et al*.^53^ proposed a scheme of CPF gate without strict strong coupling on a silicon chip. An *et al*.^54^ presented a scheme for QIP with a single photon by an input-output process with respect to bad cavities. 2009, Chen *et al*.^55^ achieved CPF gates by modifying the original idea proposed by An *et al*.^54^.

In this paper, we present a deterministic scheme for constructing a CNOT gate on a hybrid photon-atom system through the atom-cavity photon scattering. In our scheme, the control qubit is encoded on a flying photon (i.e., the two polarization states of a single photon, the right circular polarization and the left circular polarization), while the target qubit is encoded on the ground states of an atom trapped in a bad optical microcavity. We also present a deterministic scheme for constructing a Toffoli gate on a photon-atom-atom hybrid system. We use the atom-cavity systems as our quantum nodes to realize our two quantum gates. The long coherence time of the system is feasible for multi-time operations between the photon and the system and it is suitable for perfect quantum memory. These two gates work in the intermediate coupling region with bad cavities, not require strong coupling strength with good cavities, which relaxes the difficulty of their implementation in experiment. In the bad cavity limit, *κ* ≫ *g*^2^/*κ* ≫ *γ*, it is feasible for fast reading out information, and it is effective for reducing the interaction time between the photon and the atom-cavity system. Our two gates do not require any auxiliary qubits and measurements. Moreover, our schematic setup of the Toffoli gate is very simple, as only a quarter wave packet is used to interact the photon with the atom-cavity system every time, which can reduce the imperfection of the nonlinear interaction. It will be shown that high average fidelities and efficiencies can be achieved for these gates with the intermediate coupling between the atom and the cavity region.

## Results

### The single-photon input-output process

**Figure 1:**
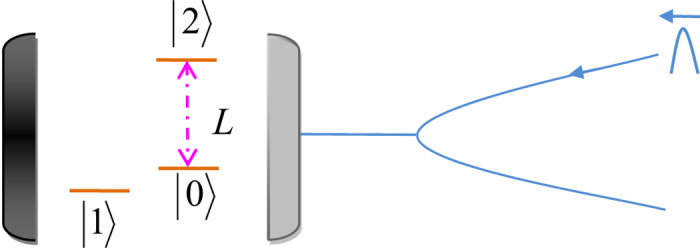
The optical transitions of an atom trapped in a single-sided optical cavity with circularly polarized lights. The *left* wall of the cavity is perfectly reflective and the *right* one is partially reflective. |0〉, |1〉, and |2〉 represent the two ground states and the one excited state of the atom, respectively. *L* represents the left circularly polarized photon.

Let us consider an atom which has two ground states |0〉 and |1〉 and an excited state |2〉 trapped in a single-sided optical cavity, shown in Fig. 1. The cavity considered here is one side wall perfectly reflective and the other side wall partially reflective^41^. The left-circularly *L* polarized cavity mode couples the transition |0〉 ↔ |2〉 (for example, the D2 transition (6*S*_1/2_, *F* = 4, *m* = 4) → (6*P*_3/2_, *F*′ = 5, *m*′ = 5) of cesium), while it decouples the transition |1〉 ↔ |2〉 because of large detuning. Under the Jaynes-Commings model, the Hamiltonian of the whole system composed of a single cavity mode (*L* polarized) and an atom trapped in a single-sided cavity can be expressed as:


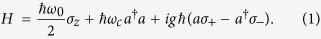


Here *a* and *a*^†^ are the annihilation and creation operators of the *L* polarized cavity mode with the frequency *ω*_*c*_, respectively. *σ*_*z*_, *σ*_+_, and *σ*_−_ are the inversion, raising, and lowering operators of the atom, respectively. *ω*_0_ is the frequency difference between the ground level |0〉 and the excited level |2〉 of the atom. *g* is the atom-cavity coupling strength, which is affected by the trapping position of the atom. The reflection coefficient of a single-photon pulse with the frequency *ω*_*p*_ injected into the optical cavity can be obtained by solving the Heisenberg- Langevin equations of motion for the internal cavity field and the atomic operators in the interaction picture^62^:


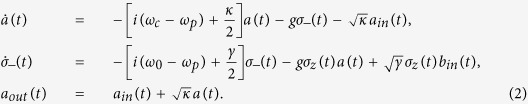


Here the one-dimensional field operator *a*_*in*_(*t*) is the cavity input operator which satisfies the commutation relation 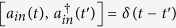. *b*_*in*_(*t*), with the commutation relation 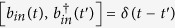, is the vacuum input field felt by the three-level atom. *a*_*out*_ is the output operator. *κ* and *γ* are the cavity damping rate and the atomic decay rate, respectively.

The atom is prepared in the ground states initially. By making *κ* sufficiently large, one can ensure that the excitation by a single-photon pulse is a weak one, and obtain the input-output relation of the cavity field^54^


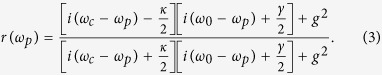


Here 
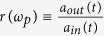
 is the reflection coefficient for the atom-cavity system. When the atom is uncoupled to the cavity or an empty cavity, that is, *g* = 0, one can obtain^62^


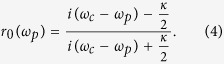


If the atom is initially prepared in the ground state |0〉, the left-circularly polarized single-photon pulse |*L*〉 will drive the transition |0〉 ↔ |2〉. The output pulse related to the input one can be expressed as |Φ_*out*_〉_*L*_ = *r*(*ω*_*p*_)|*L*〉 ≈ *e*^*iϕ*^|*L*〉. The phase shift *ϕ* is determined by the parameter values in Eq. (3). However, if the atom is initially prepared in the ground state |1〉, the left-circularly polarized single-photon |*L*〉 will only sense a bare cavity. As a result, the corresponding output governed by Eq. (4) is 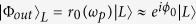, with a phase shift 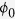 different from *ϕ*. Considering the parameters of the atom-cavity system satisfy the relationship *ω*_0_ = *ω*_*c*_ = *ω*_*p*_, the reflection coefficient can be expressed as


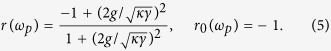


Considering a bad cavity *κ* ≫ *g*^2^/*κ* ≫ *γ* in the atom-cavity intermediate coupling region, phase shifts *ϕ* = 0 and *ϕ*_0_ = *π* from Eq. (5) can be produced.

### CNOT gate on a two-qubit hybrid system

**Figure 2:**
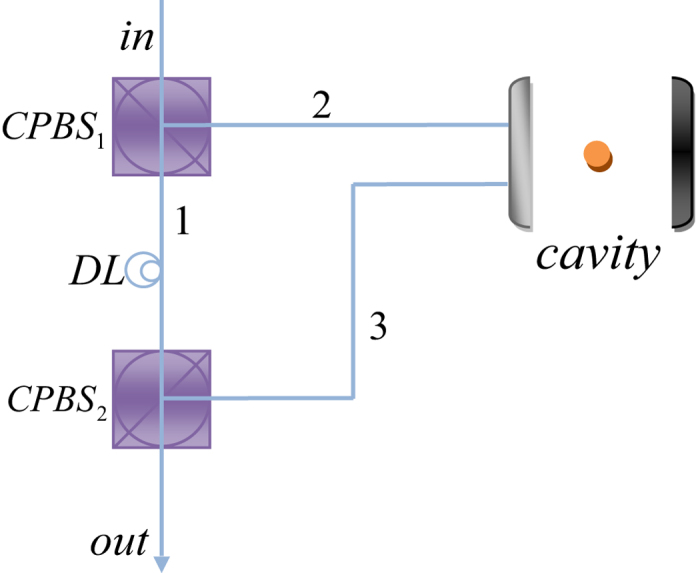
The schematic setup for a deterministic CNOT gate with a flying polarized photon as the control qubit and an atom trapped in a single-sided optical cavity as the target qubit. *CPBS*_*i*_ (*i* = 1, 2) is a circularly polarizing beam splitter which transmits the photon in the right-circular polarization |*R*〉 and reflects the photon in the left-circular polarization |*L*〉, respectively. *M* is a mirror. *DL* is a time-delay device which makes the two wavepackets coming from the paths 2 and 3 interfere with each other.

Our CNOT gate on a two-qubit hybrid system is used to complete a bit-flip on the atom trapped in the cavity when the flying photon is in the left-circular polarization |*L*〉; otherwise, it does nothing. The schematic setup for our CNOT gate is shown in Fig. 2. We will describe its principle in detail as follows.

Suppose that the initial states of the flying photon *p* and the atom *a* trapped in the single-sided cavity are


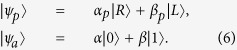


First, the flying photon is led to the device shown in Fig. 2. The circularly polarizing beam splitter *CPBS*_1_ transmits the photon in the right-circular polarization |*R*〉 to path 1 and reflects the photon in the left-circular polarization |*L*〉 to path 2. The state of the hybrid system composed of the flying photon *p* and the atom *a* is changed from |Ψ〉_0_ ≡ |*ψ*_*p*_〉 ⊗ |*ψ*_*a*_〉 to |Ψ〉_1_. Here


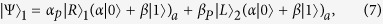


where the subscripts 1 and 2 represent the paths that the flying photon passes through. The subscript *a* represents the atom trapped in the cavity.

Second, a Hadamard operation is performed on the atom trapped in the cavity before the photon interacts with the atom-cavity system. The Hadamard operation on the atom is used to complete the transformations 
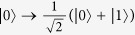
 and 
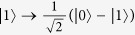
. Thus, the state of the hybrid system is changed to be


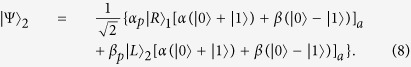


Third, the photon interacts with the atom trapped in the single-sided cavity and the state of the system becomes


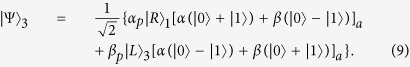


After the interaction between the flying photon and the atom trapped in the cavity, a Hadamard operation is performed on the atom again. At last, the two wavepacks split by *CPBS*_1_ reunion at *CPBS*_2_ from path 1 and path 3. The state of the system is transformed into


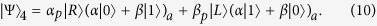


Here |Ψ〉_4_ is the objective state. One can see that the state of the atom (the target qubit) is flipped when the photon (the control qubit) is in the left-circular polarization |*L*〉; otherwise, nothing is done on the atom. That is, the schematic setup shown in Fig. 2 can be used to deterministically achieve a quantum CNOT gate on the photon-atom hybrid system by using the flying photon as the control qubit and the atom as the target qubit in principle.

### Toffoli gate on a three-qubit hybrid system

**Figure 3:**
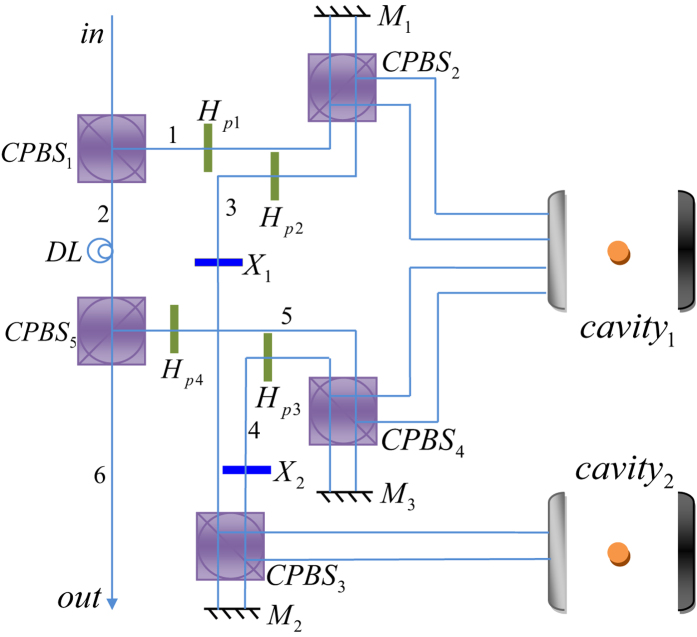
The schematic setup for constructing a deterministic Toffoli gate with the polarization of a flying photon and an atom trapped in a single-sided cavity (*cavity*_1_) as the two control qubits and anther atom trapped in another single-sided cavity (*cavity*_2_) as the target qubit. *H*_*pi*_(*i* = 1, 2, 3, 4) is a half-wave plate with the axis at 22.5° and it performs a Hadamard operation on the photon. *X*_*i*_ (*i* = 1, 2) represents a half-wave plate which performs a bit-flip operation on the photon. *M*_*i*_ (*i* = 1, 2, 3) is a mirror. *cavity*_*i*_ (*i* = 1, 2) represents the atom-cavity system.

Our Toffoli gate on a three-qubit hybrid system is used to complete a bit-flip operation on the atom trapped in *cavity*2 (the target qubit) when the polarization of the flying photon (the first control qubit) is in the left-circular polarization |*L*〉 and the atom trapped in *cavity*1 (the second control qubit) is in the state |1〉 at the same time; otherwise, it does nothing on the atom trapped in *cavity*2. The schematic setup of our Toffoli gate is shown in Fig. 3. Assume that the initial states of the flying photon qubit and the two atoms trapped in *cavity* 1 and *cavity* 2 are prepared in |*ϕ*_*p*_〉, |*ϕ*_*a*1_〉, and |*ϕ*_*a*2_〉, respectively. Here,


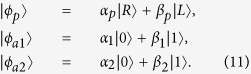


The principle of our Toffoli gate can be described in detail as follows.

First, the photon is led into our device from the port *in*. *CPBS*_1_ reflects the photon in the left-circular polarization |*L*〉 to path 1 and transmits the photon in the right-circular polarization |*R*〉 to path 2. The photon passing through path 2 will not interact with the atoms trapped in cavities. After the photon passes through *CPBS*_1_, the state of the system is changed from |Φ〉_0_ ≡ |*ϕ*_*p*_〉 ⊗ |*ϕ*_*a*1_〉 ⊗ |*ϕ*_*a*2_〉 to |Φ〉_1_. Here,


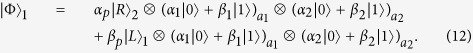


Second, a Hadamard operation is performed on the photon in path 1, and *CPBS*_2_ transmits the photon in |*R*〉 to *M*_1_ and reflects the photon in |*L*〉 to *cavity* 1. Here the Hadamard operation on the photon completes the transformations 
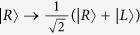
 and 
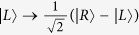
. Subsequently, the flying photon interacts with the atom trapped in *cavity* 1. After the interaction, the two components of the photon reunion at *CPBS*2. Also, a Hadamard operation and a bit-flip operation *σ*_*x*_ = |*L*〉〈*R*| + |*R*〉〈*L*| are performed on the photon in path 3. The state of the whole system becomes


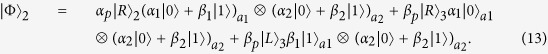


Third, one can perform a Hadamard operation on the atom trapped in *cavity* 2 and lead the photon in |*L*〉 reflected by *CPBS*_3_ to *cavity* 2 and the photon in |*R*〉 transmitted by *CPBS*_3_ to *M*_2_. The photon in |*L*〉 and the atom trapped in *cavity* 2 interact with each other. After the interaction, a Hadamard operation is performed on the atom trapped in *cavity* 2 again. The two components of the photon reunion at *CPBS*_3_. The state of the whole system is changed into


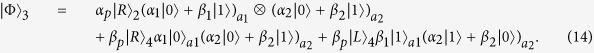


Finally, a bit-flip operation and a Hadamard operation are performed on the photon which emerges in path 4. *CPBS*_4_ transmits the photon in |*R*〉 to *M*_3_ and reflects the photon in |*L*〉 to *cavity* 1. The photon in |*L*〉 interacts with the atom trapped in *cavity* 1 again. After the interaction between the atom-cavity system and the photon, *CPBS*_4_ reflects the photon in |*L*〉 and transmits the photon in |*R*〉 to path 5. The former is reflected by *cavity* 1 and the latter is reflected by *M*_3_. A Hadamard operation is performed on the photon in path 5. At this time, the two components of the photon from paths 2 and 5 pass through *CPBS*_5_ simultaneously, and then the photon is led out of our device. The final state of the whole system composed of the flying photon and the two atoms trapped in two cavities separately can be expressed as


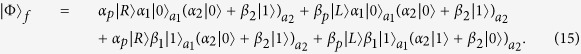


From Eq. (15), one can see that the state of the atom trapped in *cavity* 2 (the target qubit) is flipped only when the photon (the first control qubit) is in the left-circular polarization |*L*〉 and the atom trapped in *cavity* 1 (the second control qubit) is in |1〉 at the same time. That is, the schematic setup shown in Fig. 3 can achieve a quantum Toffoli gate on a photon-atom-atom hybrid system by using the flying photon and the atom in *cavity* 1 as the two control qubits and the atom in *cavity* 2 as the target qubit in a deterministic way.

## Discussion

Reiserer *et al*.^47^ exploited the atom-cavity system to complete a robust photon detection scheme experimentally. In their experiment, a single ^87^Rb atom is trapped at the center of a Fabry-Perot resonator^63^. Their experiment was completed in the experimental parameters [*g*, *κ*, *γ*]/2*π* = [6.7, 2.5, 3.0]*MHz*. In the same experimental parameters, they^49^ implemented a quantum CNOT gate that a flip of the photon is controlled by an atom trapped in a Fabry-Perot cavity. Tiecke *et al*.^48^ realized a scheme in which a single atom switches the phase of a photon and a single photon modifies the atom’s phase. Their experiment was implemented in the parameters [2*g*, *κ*, *γ*]/2*π* = [(1.09 ± 0.03) *GHz*, 25 *GHz*, 6 *MHz*]. Compared with intermediate coupling strength of the atom-cavity system, it is still challenging to realize the strong coupling strength in experiment. For obtaining shorter operation time, it is significant to realize the atom-cavity photon scattering with a bad cavity in experiment. Turchette *et al*.^51^ made a measurement on the conditional phase shifts for quantum logic in the experimental parameters [*g*, *κ*, *γ*]/2*π* = [20, 75, 2.5]*MHz*. These parameters satisfy the limit of a bad cavity *κ* ≫ *g*^2^/*κ* ≫ *γ* and an intermediate coupling region (*g* = 0.27*κ*). Based on these experimental parameters, the average fidelities of our CNOT gate and Toffoli gate are 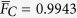 and 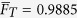, respectively. The average efficiencies of our CNOT gate and Toffoli gate are 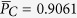 and 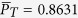, respectively. Dayan *et al*.^52^ demonstrated an intermediate atom-cavity coupling in experiment. In their experiment, a Cs atom is trapped in a microtoroidal resonator. They gave a set of parameters [*g*, *κ*, *γ*]/2*π* = [70,(165 ± 15), 2.6]*MHz*. The probe laser can be swept continuously over a range Δ = *ω*_*p*_ − *ω*_*c*_ = ±400 MHz and the atom-cavity detuning *ω*_0_ − *ω*_*c*_ = 0 can be obtained. The parameters in their experiment satisfy the requirements of a bad cavity and an intermediate coupling regime (*g* = 0.38*κ*). Based on these experimental parameters, the average fidelities of our CNOT gate and Toffoli gate are 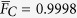 and 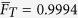, respectively. The average efficiencies of our CNOT gate and Toffoli gate are 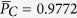 and 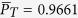, respectively. The analyses above show that the average fidelities and the averages efficiencies of our two gates can remain high values in the intermediate coupling region with a bad cavity.

In contrary to the CNOT scheme presented by Bonant *et al*.^35^, in which a confined electron spin in a QD trapped in a cavity acts as a control qubit and the spin of the photon acts as a target qubit, we use a flying photon as a control qubit and use an atom trapped in an cavity as a target qubit. Our scheme is different from the CNOT scheme proposed by Reiserer *et al*.^49^, in which an atom trapped in a cavity acts as a control qubit and the polarization state of the photon acts as a target qubit. In our scheme, the two different polarizations of the photon are split by the CPBS before the photon interacts with the atom-cavity system, which will reduce the difficulty of the experiment. Our scheme is also different from the work by Su *et al*.^59^ in which an atom trapped in an cavity acts as the control qubit and an atom trapped in another cavity acts as the target qubit with an auxiliary atom qubit and measurements on the auxiliary qubit and the photon.

In summary, we have proposed two schemes for constructing a deterministic CNOT gate and a deterministic Toffoli gate on photon-atom hybrid systems, respectively, by utilizing the nonlinear interaction between the flying photon and the atom-cavity system and some linear optical elements. For our CNOT gate, the control qubit is encoded on the flying photon and the target qubit is encoded on the atom trapped in the cavity. For our Toffoli gate, the control qubits are encoded on the flying photon and an atom trapped in one cavity and the target qubit is encoded on an atom trapped in another cavity. The quantum circuits of our two gates are very simple. They do not need any auxiliary qubit and measurements to complete the CNOT and Toffoli gates on photon-atom hybrid systems. Our two schemes can work in the atom-cavity intermediate coupling region with bad cavities. The atom-cavity system working in the intermediate coupling region is achieved in experiment^51^,^52^. The ratio of coupling strength to dissipation factors 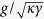 affects the fidelities and efficiencies of our gates a little. Our calculations show that even in a worst condition or a reasonable experimental condition, the average of fidelities and the average efficiencies of our two gates can remain high values. What’s more, there exist experimental parameters that satisfy the requirements in this work.

### Methods Fidelities and efficiencies of the gates

The nonlinear interaction between the single photon and the atom-cavity system produces a phase shift between the output photon and the input photon. Utilizing this shift and some linear optical elements we construct a CNOT gate and a Toffoli gate on photon-atom and photon-atom-atom hybrid quantum systems, respectively. In the process of constructing these two universal quantum hybrid gates, we set 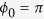 and *ϕ* = 0. In this ideal case, the hybrid quantum gates are deterministic, and the fidelity and the efficiency are 100% for each gate. However, the phase shift 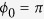 is an exact value when *g* = 0, while the phase shift *ϕ* = 0 is an approximate value when *κ* ≫ *g*^2^/*κ* ≫ *γ*. It is a function of 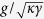, which is decided by the experimental condition. Considering the realistic condition, we will calculate the fidelities of our quantum gates to show their performance. The fidelity is defined as *F* = |〈Ψ_*r*_|Ψ_*i*_〉|^2^. Here |Ψ_*r*_〉 and |Ψ_*i*_〉 are final states of the hybrid quantum system in our schemes for quantum gates in the realistic condition and the ideal condition, respectively.

The fidelity of our CNOT gate is expressed as


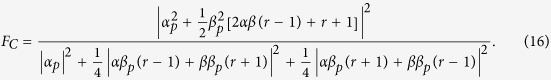


The coefficients of the system can be expressed as *α*_*p*_ = cos *φ*, *β*_*p*_ = sin *φ*, *α* = cos *θ*, and *β* = sin *θ*. The average fidelity of the CNOT gate is


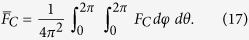


**Figure 4:**
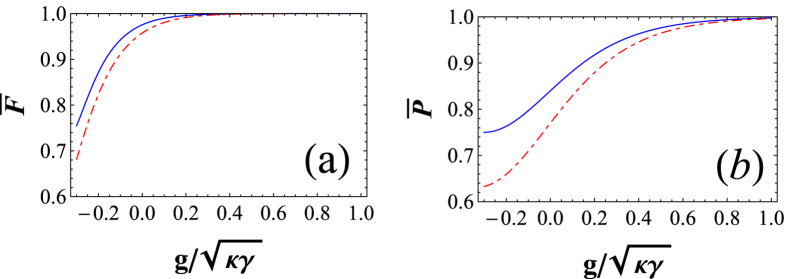
(**a**) Average fidelity 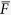 of our CNOT gate on a two-qubit hybrid system (solid line) and that of our Toffoli gate on a three-qubit hybrid system (dashed line) vs 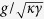 on a logarithmic scale. (**b**) Average efficiency 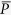 of our CNOT gate on a two-qubit hybrid system (solid line) and that of our Toffoli gate on a three-qubit hybrid system (dashed line) vs 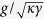 on a logarithmic scale.

The relationship between the average fidelity of our CNOT gate and 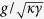 on a logarithmic scale is shown in Fig. 4(a) with the solid line. For our Toffoli gate on a three-qubit hybrid system, its fidelity is


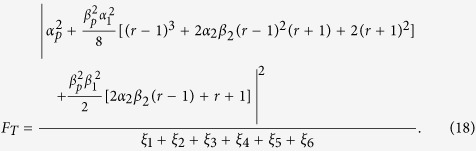


Here *ξ*_1_ = |*α*_*p*_|^2^, 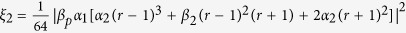, 
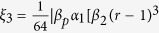

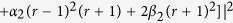
, 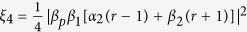, 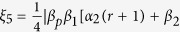

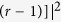
, and 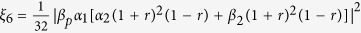. The coefficients of the system can be expressed as *α*_*p*_ = cos *φ*, *β*_*p*_ = sin *φ*, *α*_1_ = cos *θ*, *β*_1_ = sin *θ*, *α*_2_ = cos *η*, and *β*_2_ = sin *η*. The average fidelity of our Toffoli gate is


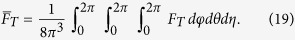


The dashed line in Fig. 4(a) shows the relationship between the average fidelity of our Toffoli gate and 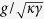 on a logarithmic scale.

The efficiency of a quantum gate is defined as 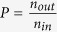, where *n*_*out*_ is the number of the photons coming out of the device and *n*_*in*_ is the number of the photons led into the device. The efficiency of our CNOT gate is


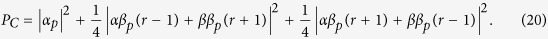


The average efficiency of our CNOT gate is


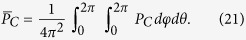


The relationship between the average efficiency of our CNOT gate and 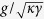 on a logarithmic scale is shown in Fig. 4(b) with the solid line.

The efficiency of our Toffoli gate is


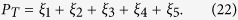


We can also calculate the average efficiency of our Toffoli gate


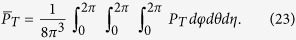


The dashed line in Fig. 4(b) shows the relationship between the average efficiency of our Toffoli gate and 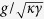 on logarithmic scale. From Fig. 4(a,b), one can see that the average fidelities and average efficiencies of these two universal quantum gates are affected by the cooperativity *C* (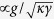) of the atom-cavity system. The average fidelities are relatively sensitive to the cooperativity when 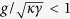 (0 on a logarithmic scale) and they are faintly affected by cooperativity when 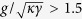 (0.17 on a logarithmic scale). If 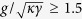, which is not a difficult experimental requirement, the average fidelities of our CNOT and Toffoli gates can be higher than 0.9949 and 0.9896, respectively. The average efficiencies are relatively sensitive to the cooperativity when 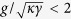 (0.3 on a logarithmic scale) and they are faintly affected by the cooperativity when 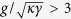 (0.48 on a logarithmic scale). If 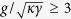, which is not a difficult experimental requirement, the average efficiencies of our CNOT and Toffoli gates can be higher than 0.9737 and 0.9609, respectively.

Except for the cooperativity *C*, some other realistic losses and imperfections, that would affect the fidelities and the efficiencies of our schemes, should be taken into account. The mismatching of spatial mode between cavity and the input photon, the quality of atomic state preparation and rotation will affect both of the fidelities and the efficiencies of our schemes^49^. The fidelities of our schemes will be also affected by the small probability of more than one photon in the input laser pulses^49^. The efficiencies can be also affected by the stability of difference between the cavity resonance and the frequency of the input photon and the imperfect absorption losses of the mirror of the cavity^49^. In our scheme, as only one polarization of a photon is injected to the atom-cavity system and the two polarizations are split by the *CPBS*, the precise timing of the arrival times from different photon paths is required in the realistic experiment.

## Additional Information

**How to cite this article**: Wang, G.-Y. *et al*. Universal quantum gates for photon-atom hybrid systems assisted by bad cavities. *Sci. Rep*. **6**, 24183; doi: 10.1038/srep24183 (2016).
